# Exploring the association between use of gonadotropin releasing hormones agonists and prostate cancer diagnosis per se and diabetes control in men with type 2 diabetes mellitus: a nationwide, population-based cohort study

**DOI:** 10.1186/s12885-021-08941-y

**Published:** 2021-11-22

**Authors:** E. Lin, Hans Garmo, Mieke Van Hemelrijck, Jan Adolfsson, Pär Stattin, Björn Zethelius, Danielle Crawley

**Affiliations:** 1 School of Cancer and Pharmaceutical Sciences, Translational Oncology and Urology Research (TOUR), King’s College London, 3rd Floor Bermondsey Wing, Guy’s Hospital, London, SE1 9RT UK; 2 Department of Surgical Sciences, Uppsala University, Uppsala, Sweden; 3 Department of Clinical Science, Intervention and Technology, Karolinska Institute, Stockholm, Sweden; 4 Department of Public Health/Geriatrics, Uppsala University, Uppsala, Sweden

**Keywords:** Prostate cancer diagnosis, Gonadotropin-releasing hormone agonists, Diabetes

## Abstract

**Background:**

Gonadotropin Releasing Hormones agonists (GnRH), which are first line treatment for metastatic prostate cancer (PCa), increase risk of type 2 diabetes mellitus (T2DM). This study aims to quantify the association of use of GnRH with diabetes control in PCa men with T2DM.

**Methods:**

Nationwide population-based cohort study in the Swedish National Diabetes Register and Prostate Cancer data Base Sweden 4.1, on the association between GnRH and diabetes control in T2DM men with PCa by comparing T2DM men with PCa vs. without PCa, as well as comparing T2DM men with PCa on or not on GnRH. The primary exposure was use of GnRH. Worsening diabetes control was the primary outcome, defined as: 1) HbA1c rose to 58 mmol/mol or higher; 2) HbA1c increase by 10 mmol/mol or more; 3) Start of antidiabetic drugs or switch to insulin. We also combined all above definitions. Cox proportional hazards regression was used to analyze the association.

**Results:**

There were 5714 T2DM men with PCa of whom 692 were on GnRH and 28,445 PCa-free men with T2DM with similar baseline characteristics. Diabetes control was worse in men with GnRH vs. PCa-free men (HR: 1.24, 95% CI: 1.13–1.34) as well as compared with PCa men without GnRH (HR:1.58, 95% CI: 1.39–1.80), when we defined the worsening control of diabetes by combining all definitions above.

**Conclusion:**

Use of GnRH in T2DM men with PCa was associated with worse glycemic control. The findings highlight the need to closely monitor diabetes control in men with T2DM and PCa starting GnRH.

## Background

Prostate cancer (PCa) is the most frequently diagnosed cancer in men in Europe, with approximately 450, 000 new cases in 2018, accounting for 24% of all newly diagnosed cancers [1]. While, in 2019, about 59 million people in Europe had a diagnosis of type two diabetes (T2DM) [2]. Thus, PCa and T2DM are common conditions that may occur concurrently in the same man [3]. Few studies have assessed the association of PCa and its hormonal treatment with diabetes control in men with pre-existing T2DM.

Gonadotropin-releasing hormone agonists (GnRH) are first line treatment for metastatic PCa and are also widely used in conjunction with radiotherapy in locally advanced PCa as both neoadjuvant and adjuvant therapy [4]. GnRH have a range of side effects, including a metabolic like syndrome [5]. An association between use of GnRH and T2DM has been demonstrated in many observational studies, and it is established that GnRH lead to increased insulin resistance and risk of diabetes [6–8]. In 2010, the Food and Drug Administration required labelling of all GnRH with a warning of an increased risk of diabetes and cardiovascular diseases [9]. However, few studies have examined the effect of GnRH on diabetes control in men with pre-existing T2DM.

Our aim was to investigate the association between use of GnRH and diabetes control, both in terms of glycemic control and changes in antidiabetic drugs, in men with T2DM and PCa.

## Methods

### Data source

Prostate Cancer data Base Sweden (PCBaSe) 4.1 is a database based on the National Prostate Cancer Register (NPCR) of Sweden, which contains information on 98% of men in Sweden diagnosed with PCa between 1998 and 2016 compared with The Cancer Registry to which reporting is mandated [10]. In PCBaSe 4.1, men in NPCR were linked to other nationwide databases, including National Patient Register, Longitudinal integrated database for health insurance and labor market studies, Swedish National Cancer Register and other nationwide registers [10] by use of the unique personal identity number of all residents. We obtained data on PCa characteristics, co-morbidities, civil status and educational level from PCBaSe 4.1 [10]. We also collected prescribed medications data from the National Prescribed Drug Register (PDR) which was established in July 2005 [11]. The PDR contains information of all prescribed drugs dispensed at pharmacies covering the whole population of Sweden.

Moreover, information on diabetic conditions was retrieved through a linkage between PCBaSe 4.1 and the National Diabetes Registry (NDR) which was initiated in 1996 and has engaged the participation of both hospitals and primary care. This register contains detailed data on demographics, smoking, diabetes duration, treatment modalities, risk factors and diabetes complications and it currently includes most of T2DM patients age 18 and older in Sweden [12, 13].

The study population included men diagnosed with T2DM, according to NDR, amongst men included in PCBaSe 4.1 between 2006 and 2016.

### Study population

**Fig. 1:**
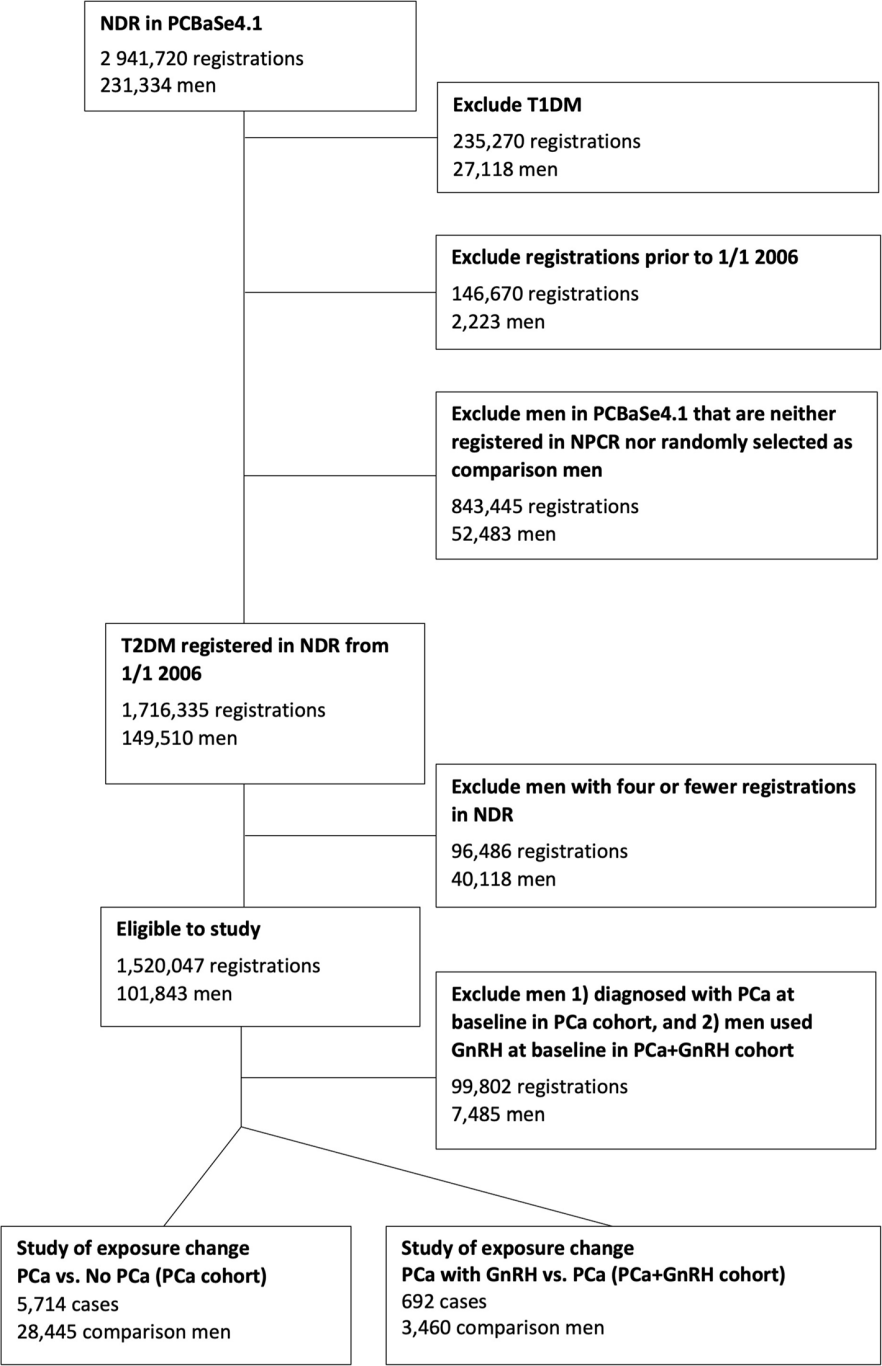
Patient inclusion and exclusion flowchart. Figure 1. This figure illustrated the study design and patient selection process. We included men diagnosed with type 2 diabetes mellitus (T2DM), according to the National Diabetes Registry (NDR), amongst men included in Prostate Cancer data Base Sweden (PCBaSe) 4.1 in 2006–2016 and created two cohorts – “Prostate cancer (PCa) + Gonadotropin-releasing hormone agonists (GnRH) exposure cohort” and “GnRH exposure cohort”. 5714 men with PCa and 28,445 PCa-free men were included in the PCa + exposure cohort. The GnRH exposure cohort contained 692 PCa men who started GnRH after PCa diagnosis and 3460 PCa men not using GnRH as comparison

To investigate the association of use of GnRH and a PCa diagnosis per se with diabetes control separately, we created two cohorts of men with a diagnosis of T2DM – “PCa+GnRH exposure cohort” and “GnRH exposure cohort” (Fig. 1).

In the PCa + GnRH exposure cohort, we included men with at least four registrations of data in NDR who were diagnosed with PCa on GnRH or not on GnRH after their third NDR-registration. Date of PCa diagnosis was considered as start of follow up. For each man with PCa in our study, five PCa-free men with T2DM were randomly selected from the NDR, matched on average duration between NDR visits and number of previous NDR registrations. Start of follow-up for these men was ‘inherited’ from the corresponding man with a PCa diagnosis.

The GnRH exposure cohort consisted of men with PCa and T2DM who initiated use of GnRH after the third registered date in the NDR. Date of the first filled prescription for GnRH registered in the PDR was considered start of follow up. As a comparison, we selected five men with PCa not on GnRH randomly from the NDR, matched on average duration between NDR visits and the number of previous NDR registrations. Start of follow up for these men was ‘inherited’ from the corresponding man treated with GnRH.

When we defined the GnRH exposure in both cohorts, we excluded the prescriptions that were part of a radical radiotherapy treatment by combining information on usage of GnRH (ATC-code L02AE) from PDR and information on duration of neoadjuvant and adjuvant GnRH treatment in relation to radical radiotherapy recorded in NPCR. In a study on a similar group of men conducted by George et al. (2020), good adherence to GnRH was shown [14]. Longer injection intervals and higher risk groups showed an increased adherence in men on primary GnRH [14]. Therefore, we assumed that the use of GnRH in our study referred to lifelong treatment.

In our study, only a few men underwent orchiectomy receiving GnRH were included in both cohorts. We performed a sensitivity analysis to assess whether orchiectomy was affecting our study findings. Besides, we used the NDR registrations in the period between 2006 and 2017. The novel androgen axis targeting agents, such as enzalutamide and abiraterone, were introduced but with a high price since 2015. The National Prescribed Drug Register (PDR) included the data on these agents since 2017. There was limited number of men used these agents in our study. Therefore, the effect of these agents on the association between GnRH and glycemic control can be ignored.

### Exposures

The primary exposure was use of GnRH. In addition, we also used information on PCa diagnosis and PCa risk categories from NPCR and PDR. According to the National Comprehensive Cancer Network (NCCN) guideline, there are five risk categories for PCa: 1) low-risk category: T1 or T2a stage, PSA < 10 ng/mL and Gleason score 6; ii) intermediate-risk category: T2b or T2c stage, 10 ng/mL, PSA < 20 ng/mL, or Gleason score 7; iii) high-risk category: T3a or T4 stage, PSA ≥ 20 ng/mL, or Gleason score ≥ 8; iv) regional metastases category: any T, N1 and M0 stage; v) distant metastases category: any T or N and M1 stage [15].

### Outcomes

The primary outcome was worsening of diabetes control based on information collected as part of the longitudinal follow-up in the NDR. According to the National Institute for Health and Care Excellence (NICE) guidance, the definition of worsening diabetes control includes [16]:

1. HbA1c rose to 58 mmol/mol or higher (for men not already > 58 mmol/mol at baseline)
2. HbA1c was 10 mmol/mol higher than the baseline measurement.
3. Commencement of antidiabetic drugs or switch to insulin (for men not already on insulin at baseline).

We also combined the above criteria. For men whose HbA1c was less than 58 mmol/L and who did not use insulin at the baseline, we included all of the above definitions with whichever occurred first as the combination event. For men whose HbA1c was over 58 mmol/L and/or who used insulin at the baseline, we used the remaining definitions, with whichever came first as the combination event.

### Data analysis

We used the NDR registrations in the period 1/12006 to 31/122017. The baseline measurements for a participant were based on the three last NDR-registrations prior to the start of follow up. Missing data was imputed using last observation carried forward, i.e., if the last observation in NDR was missing then information was taken from the second last, if that was also missing it was retrieved from the third last NDR registration. If all the last three NDR observations were missing, then data was classified as missing.

Time to event was defined as the time from start of follow up to the first date of worsening diabetes control or last observation in NDR, whichever came first. Hazard ratios (HR) and 95% confidence interval (CI) for worsening of diabetes control as defined above were obtained using Cox proportional hazards regression models. All models were adjusted for age at PCa onset, duration of T2DM, education level, civil status, the Charlson Comorbidity Index (CCI), smoking habits, physical activity and body mass index (BMI). Cumulative incidence of worsening T2DM control was presented using Kaplan-Meier curves.

All data management was performed with Statistical Analysis Systems release 9.4 (SAS Institute, Cary, NC) and R 3.5.2 (R Foundation for Statistical Computing). The study has been approved by The Research Ethics Board at Uppsala University, Sweden.

## Results

5714 men with PCa and 28,445 PCa-free men were included in the PCa + GnRH exposure cohort. The GnRH exposure cohort contained 692 PCa men who started GnRH after PCa diagnosis and 3460 PCa men not using GnRH as comparison. Baseline characteristic for age, education level, civil status, CCI, smoking habits, BMI, physical activity, and T2DM status (including T2DM duration, T2DM treatments and HbA1c) were similar between PCa men or PCa men with GnRH and the comparison groups in both PCa + GnRH exposure cohort and GnRH exposure cohort, respectively (Table 1).

**Table 1:** Baseline characteristics of men in NDR diagnosed with prostate cancer and/or used GnRH between 2006 and 2016 and their matched comparison

	PCa + GnRH exposure cohort		GnRH exposure cohort	
	PCa men (*N* = 5714)	No PCa men (*N* = 28,445)	PCa using GnRH men (*N* = 692)	PCa without using GnRH men (*N* = 3460)
***Patients characteristics***				
**Age (year), No. (%)**				
< 60	233 (4.1)	1071 (3.8)	8 (1.2)	60 (1.7)
60–69	1886 (33.0)	8034 (28.2)	101 (14.6)	805 (23.3)
70–79	2608 (45.6)	12,984 (45.6)	304 (43.9)	1730 (50.0)
80+	987 (17.3)	6356 (22.3)	279 (40.3)	865 (25.0)
**Education level, No. (%)**				
Low	2340 (41.0)	12,231 (43.0)	308 (44.5)	1333 (38.5)
Middle	2755 (48.2)	13,172 (46.3)	297 (42.9)	1678 (48.5)
High	570 (10.0)	2686 (9.4)	82 (11.8)	432 (12.5)
Missing	49 (0.9)	356 (1.3)	5 (0.7)	17 (0.5)
**Civil status, No. (%)**				
Married	3708 (64.9)	17,582 (61.8)	444 (64.2)	2248 (65.0)
Not married (+Divorced/Widower/missing)	2006 (35.1)	10,863 (38.2)	248 (35.8)	1212 (35.0)
**CCI, No. (%)**				
0	2413 (42.2)	10,319 (36.3)	197 (28.5)	942 (27.2)
1	1348 (23.6)	6494 (22.8)	174 (25.1)	1014 (29.3)
2	765 (13.4)	4246 (14.9)	109 (15.8)	538 (15.5)
3+	1188 (20.8)	7386 (26.0)	212 (30.6)	966 (27.9)
**Smoking, No. (%)**				
No	4581 (80.2)	22,273 (78.3)	524 (75.7)	2683 (77.5)
Yes	553 (9.7)	2912 (10.2)	51 (7.4)	267 (7.7)
Missing	580 (10.2)	3260 (11.5)	117 (16.9)	510 (14.7)
**Times of at least 60 min physical activity in 7 days, No. (%)**				
Daily	573 (10.0)	3452 (12.1)	98 (14.2)	380 (11.0)
3–5 times a week	500 (8.8)	2486 (8.7)	69 (10.0)	268 (7.7)
1–2 times a week	902 (15.8)	4261 (15.0)	96 (13.9)	503 (14.5)
Less than once a week	1016 (17.8)	4786 (16.8)	104 (15.0)	629 (18.2)
Never	1531 (26.8)	6919 (24.3)	140 (20.2)	784 (22.7)
Missing	1192 (20.9)	6541 (23.0)	185 (26.7)	896 (25.9)
**BMI (kg/m2), No. (%)**				
< 25	969 (17.0)	4668 (16.4)	111 (16.0)	610 (17.6)
25–29	2509 (43.9)	11,845 (41.6)	291 (42.1)	1507 (43.6)
30–34	1342 (23.5)	6677 (23.5)	150 (21.7)	731 (21.1)
35–39	353 (6.2)	1961 (6.9)	39 (5.6)	187 (5.4)
40+	91 (1.6)	612 (2.2)	15 (2.2)	52 (1.5)
Missing	450 (7.9)	2682 (9.4)	86 (12.4)	373 (10.8)
**Number of visits, No. (%)**				
3–9	3794 (66.4)	18,879 (66.4)	504 (72.8)	2520 (72.8)
10–19	1520 (26.6)	7578 (26.6)	151 (21.8)	755 (21.8)
20–29	315 (5.5)	1567 (5.5)	29 (4.2)	145 (4.2)
30+	85 (1.5)	421 (1.5)	8 (1.2)	40 (1.2)
***T2DM status***				
**Duration of T2DM (Years), No. (%)**				
< 10	2751 (48.1)	12,755 (44.8)	320 (46.2)	1703 (49.2)
10–19	1939 (33.9)	10,149 (35.7)	222 (32.1)	1139 (32.9)
20–29	530 (9.3)	3123 (11.0)	78 (11.3)	310 (9.0)
30+	158 (2.8)	921 (3.2)	23 (3.3)	90 (2.6)
Missing	336 (5.9)	1497 (5.3)	49 (7.1)	218 (6.3)
**HbA1c (mmol/mol), No. (%)**				
< 40	330 (5.8)	1484 (5.2)	63 (9.1)	221 (6.4)
40–57	3673 (64.3)	16,834 (59.2)	434 (62.7)	2181 (63.0)
58–69	1093 (19.1)	6045 (21.3)	119 (17.2)	642 (18.6)
70–79	324 (5.7)	2122 (7.5)	37 (5.3)	187 (5.4)
80–89	131 (2.3)	929 (3.3)	14 (2.0)	98 (2.8)
90+	87 (1.5)	612 (2.2)	12 (1.7)	61 (1.8)
Missing	76 (1.3)	419 (1.5)	13 (1.9)	70 (2.0)
**Primary treatment of T2DM, No. (%)**				
Insulin	3420 (59.9)	10,246 (36.0)	237 (34.2)	1080 (31.2)
Oral Hypoglycaemics	426 (7.5)	2330 (8.2)	46 (6.6)	276 (8.0)
Diet controlled	1868 (32.7)	15,869 (55.8)	409 (59.1)	2104 (60.8)
***PCa status***				
**PCa diagnosis, No. (%)**				
No PCa	–	28,445 (100.0)	–	–
PCa	5714 (100.0)	–	692 (100.0)	3460 (100.0)
**Using GnRH, No. (%)**				
No PCa	–	28,445 (100.0)	–	–
No	4274 (74.8)	–	–	3460 (100.0)
Yes	1400 (25.2)	–	692 (100.0)	–
**PCa risk category, No. (%)**				
No PCa	–	28,445 (100.0)		
Low risk	1122 (19.8)	–	145 (21.0)	1437 (41.5)
Intermediate risk	1838 (32.2)	–	229 (33.1)	1272 (36.8)
High risk	1531 (26.8)	–	232 (33.5)	533 (15.4)
Regional metastasises	389 (6.8)	–	42 (6.1)	56 (1.6)
Distance metastasises	650 (11.4)	–	32 (4.6)	39 (1.1)
Missing data	184 (3.2)	–	12 (1.7)	123 (3.6)

PCa denotes Prostate Cancer; T2DM: Type 2 diabetes mellitus; BMI: body mass index; CCI: Charlson Comorbidity Index

### PCa + GnRH exposure cohort

When we combined all above definitions of worsening control of diabetes, we found a positive association between use of GnRH and diabetes control (HR: 1.24, 95% CI: 1.13–1.34), compared with men without PCa, but no increased risk was seen for those with PCa not using GnRH (HR: 0.98, 95%CI: 0.93–1.03) (Table 2). However, no association of PCa diagnosis (all risk categories combined) with diabetes control was found, compared with PCa-free men with T2DM (HR:1.04, 95%CI: 0.99–1.08) (Table 2). Interestingly, the risk of worsening diabetes control was increased in men with advanced PCa compared with men without PCa. The HR for worsened diabetes control was 1.28 (95% CI: 1.10–1.50) for men with regional metastatic PCa, and 1.23 (95% CI: 1.09–1.40) in men with distant metastases, as compared with PCa-free men. In these men with advanced PCa, more than half of them used GnRH (54.8% in regional metastases group; 68.3% in distance metastases group). Additionally, Table 2 also showed results of the association between use of GnRH and PCa diagnosis and worsening control of diabetes which was defined by the changes of HbA1c or the escalation of antidiabetic drugs.

**Table 2:** HR and 95%CI for change in diabetes control in PCa + GnRH exposure cohort

	HbA1c rose to 58 mmol/mol ^**a**^		HbA1c increased 10 mmol/mol ^**b**^		Change of T2DM drugs ^**c**^		Combination of all definitions	
	HR	95%CI	HR	95%CI	HR	95%CI	HR	95%CI
***Crude model***								
**Using GnRH, n (%)**								
No PCa	1.00	ref.	1.00	ref.	1.00	ref.	1.00	ref.
No	0.94	(0.88–1.00)	1.02	(0.96–1.08)	0.94	(0.84–1.04)	0.96	(0.92–1.01)
Yes	1.20	(1.08–1.33)	1.38	(1.26–1.51)	1.13	(0.95–1.34)	1.21	(1.11–1.31)
**PCa diagnosis**								
No	1.00	Ref.	1.00	ref.	1.00	ref.	1.00	ref.
Yes	0.99	(0.94–1.05)	1.10	(1.04–1.15)	0.98	(0.89–1.07)	1.02	(0.97–1.06)
**PCa risk category**								
No PCa	1.00	ref.	1.00	ref.	1.00	ref.	1.00	ref.
Low risk	0.91	(0.81–1.02)	0.99	(0.89–1.10)	1.15	(0.98–1.36)	0.97	(0.88–1.06)
Intermediate risk	0.97	(0.89–1.06)	1.00	(0.92–1.09)	0.94	(0.81–1.10)	1.00	(0.93–1.07)
High risk	0.95	(0.85–1.05)	1.10	(1.01–1.21)	0.85	(0.71–1.01)	0.98	(0.90–1.06)
Regional metastasises	1.25	(1.04–1.52)	1.44	(1.21–1.70)	1.06	(0.76–1.49)	1.25	(1.07–1.46)
Distance metastasises	1.31	(1.11–1.53)	1.55	(1.35–1.78)	0.81	(0.58–1.13)	1.21	(1.06–1.38)
Missing data	1.04	(0.77–1.40)	1.06	(0.82–1.38)	1.34	(0.87–2.06)	0.95	(0.75–1.20)
***Adjusted model*** ^**d**^								
**GnRH, n (%)**								
No PCa	1.00	ref.	1.00	ref.	1.00	ref.	1.00	ref.
No	0.97	(0.91–1.03)	1.06	(1.00–1.12)	0.94	(0.84–1.04)	0.98	(0.93–1.03)
Yes	1.23	(1.11–1.36)	1.41	(1.28–1.54)	1.20	(1.01–1.42)	1.24	(1.13–1.34)
**PCa diagnosis**								
No	1.00	ref.	1.00	ref.	1.00	ref.	1.00	ref.
Yes	1.03	(0.97–1.08)	1.14	(1.08–1.19)	0.99	(0.90–1.09)	1.04	(0.99–1.08)
**PCa risk category**								
No PCa	1.00	ref.	1.00	ref.	1.00	ref.	1.00	ref.
Low risk	0.95	(0.84–1.06)	1.04	(0.94–1.15)	1.12	(0.95–1.32)	0.99	(0.90–1.08)
Intermediate risk	1.01	(0.92–1.10)	1.05	(0.97–1.14)	0.95	(0.81–1.10)	1.02	(0.95–1.10)
High risk	0.97	(0.87–1.07)	1.13	(1.03–1.24)	0.90	(0.75–1.07)	1.00	(0.92–1.08)
Regional metastases	1.29	(1.07–1.56)	1.47	(1.24–1.74)	1.14	(0.82–1.59)	1.28	(1.10–1.50)
Distance metastases	1.36	(1.16–1.60)	1.59	(1.38–1.82)	0.88	(0.63–1.22)	1.23	(1.09–1.40)
Missing data	0.96	(0.71–1.29)	1.01	(0.78–1.31)	1.22	(0.80–1.88)	0.92	(0.73–1.16)

a. Men with a HbA1c over 58 mmol/l and men without HbA1c data at baseline were excluded

b. Men without HbA1c date at baseline were excluded

c. Men using insulin at the baseline were excluded

d. This model was adjusted for age at PCa diagnosis, duration of T2DM, education level, CCI, civil status, smoking habits, physical activity and BMI

**Fig. 2:**
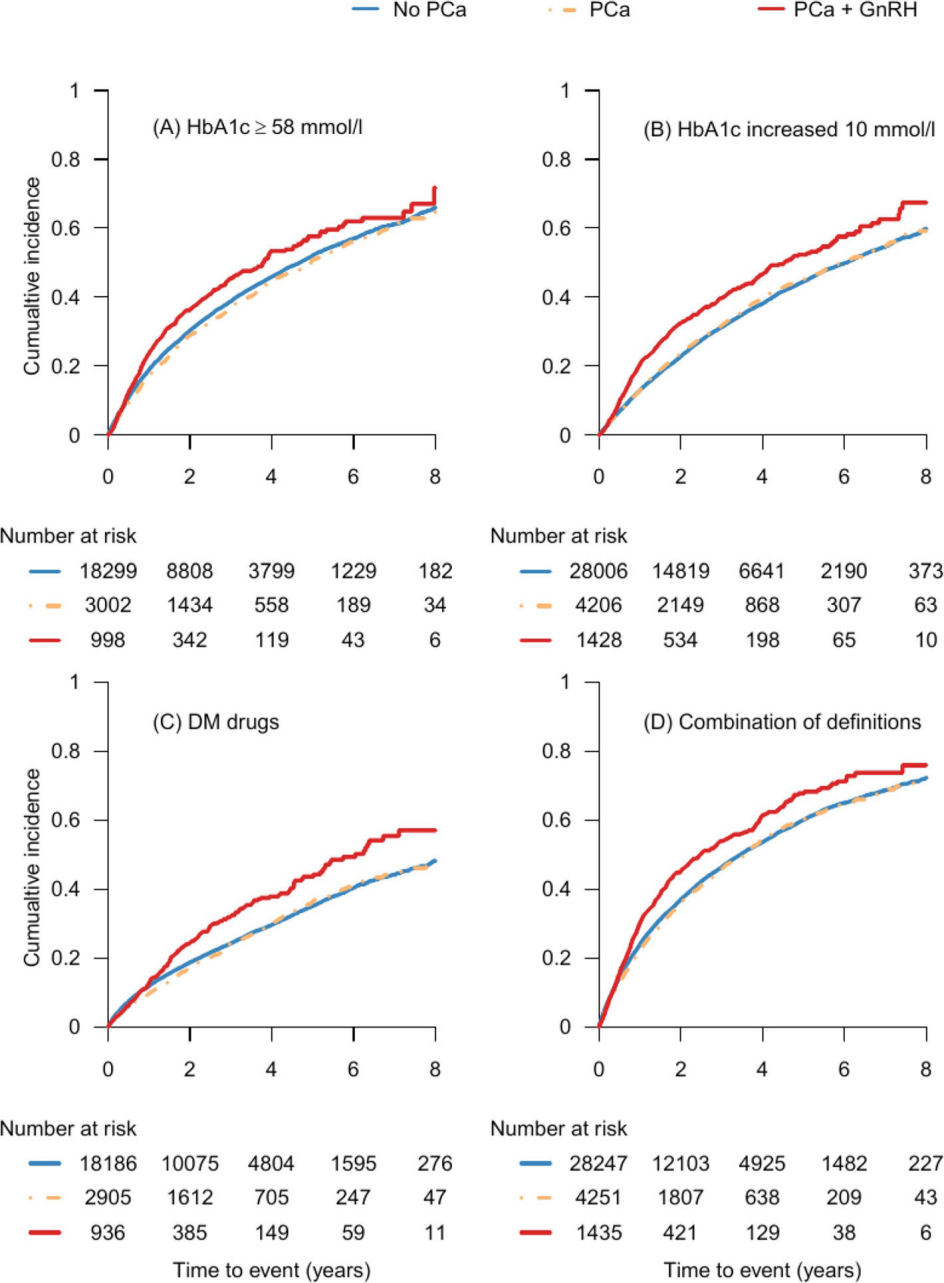
Cumulative incidence of worsening T2DM control in T2DM men by PCa status in PCa + GnRH exposure cohort ^**a.**^ Figure 2. In this figure, we found that, in the PCa + GnRH exposure cohort, men receiving GnRH had a higher cumulative incidence for worsening diabetes control, compared with PCa free men. The changes in the HbA1c measurements (Fig. 2-(a), Fig. 2-(b)) occurred earlier and more obviously than the addition of new antidiabetic medications (Fig. 2-(c)). When we combined the criteria to identify the event in Fig. 2-(a), Fig. 2-(b) and Fig. 2-(c) to create the combination event, we found that cumulative incidence of combination event is higher in PCa men with GnRH compared with men without PCa (Fig. 2-(d)). In Fig. 2-(a), we excluded men with a HbA1c over 58 mmol/l and men without HbA1c data at baseline. In Fig. 2-(b), men without HbA1c data at baseline were excluded. Men using insulin and men without antidiabetic drugs at the baseline were excluded in Fig. 2-(c)

Only 16 men underwent orchiectomy prior to receiving GnRH, resulting in no changes in the previous findings when excluding these men (Results not shown). Men receiving GnRH had a higher cumulative incidence for worsening diabetes control, compared with PCa free men (Fig. 2).

### GnRH exposure cohort

PCa men on GnRH had a higher risk of worsening diabetes control compared with men with PCa not on GnRH, when we used combined definitions to identify the worsening diabetes control (HR:1.58, 95% CI: 1.39–1.80) (Table 3). No difference by PCa risk categories was observed (Table 3). The HR for worsening diabetes control was 1.34 (95% CI: 0.97–1.83) in regional metastatic PCa, and 1.08 (95% CI: 0.72–1.62) in men with distant metastases. When we defined the worsening control of diabetes as the changes of HbA1c or the escalation of antidiabetic drugs, similar results were observed (Table 3). Sensitivity analysis excluding 80 men who underwent orchiectomy before using GnRH did not alter the previous findings in this cohort (Results not shown), when we defined the outcomes as the changes of HbA1c and combination all definitions above.

**Table 3:** HR and 95%CI for change in diabetes control in GnRH exposure cohort

	HbA1c rose to 58 mmol/mol ^**a**^		HbA1c increased 10 mmol/mol ^**b**^		Change of T2DM drugs ^**c**^		Combination of all definitions	
	HR	95%CI	HR	95%CI	HR	95%CI	HR	95%CI
***Crude model***								
**Using GnRH, n (%)**								
No	1.00	ref.	1.00	ref.	1.00	ref.	1.00	ref.
Yes	1.60	(1.37–1.87)	1.77	(1.53–2.04)	1.14	(0.85–1.53)	1.56	(1.37–1.78)
**PCa risk category**								
Low risk	1.00	ref.	1.00	ref.	1.00	ref.	1.00	ref.
Intermediate risk	1.12	(0.98–1.29)	1.00	(0.87–1.15)	0.97	(0.76–1.23)	0.99	(0.88–1.12)
High risk	1.08	(0.90–1.29)	1.11	(0.94–1.30)	0.77	(0.56–1.07)	1.05	(0.91–1.21)
Regional metastases	1.35	(0.92–1.97)	1.38	(0.96–1.98)	0.84	(0.39–1.80)	1.33	(0.97–1.82)
Distance metastases	1.31	(0.80–2.13)	1.32	(0.84–2.06)	1.04	(0.46–2.36)	1.11	(0.75–1.65)
Missing data	0.63	(0.39–1.02)	0.83	(0.57–1.22)	0.66	(0.29–1.49)	0.81	(0.59–1.12)
***Adjusted model*** ^**d**^								
**Using GnRH, n (%)**								
No	1.00	ref.	1.00	ref.	1.00	ref.	1.00	ref.
Yes	1.56	(1.33–1.83)	1.78	(1.54–2.06)	1.21	(0.89–1.63)	1.58	(1.39–1.80)
**PCa risk category**								
Low risk	1.00	ref.	1.00	ref.	1.00	ref.	1.00	ref.
Intermediate risk	1.08	(0.94–1.26)	0.99	(0.86–1.13)	1.00	(0.78–1.28)	0.99	(0.88–1.11)
High risk	0.99	(0.83–1.20)	1.06	(0.90–1.26)	0.83	(0.60–1.16)	1.02	(0.88–1.18)
Regional metastasises	1.28	(0.87–1.87)	1.38	(0.96–1.98)	0.86	(0.40–1.85)	1.34	(0.97–1.83)
Distance metastasises	1.25	(0.77–2.04)	1.25	(0.80–1.97)	1.12	(0.49–2.55)	1.08	(0.72–1.62)
Missing data	0.56	(0.34–0.92)	0.81	(0.55–1.19)	0.63	(0.27–1.43)	0.78	(0.57–1.09)

a. Men with a HbA1c over 58 mmol/l and men without HbA1c data at baseline were excluded

b. Men without HbA1c date at baseline were excluded

c. Men using insulin at the baseline were excluded

d. This model was adjusted for age at PCa diagnosis, duration of T2DM, education level, CCI, civil status, smoking habits, physical activity and BMI

**Fig. 3:**
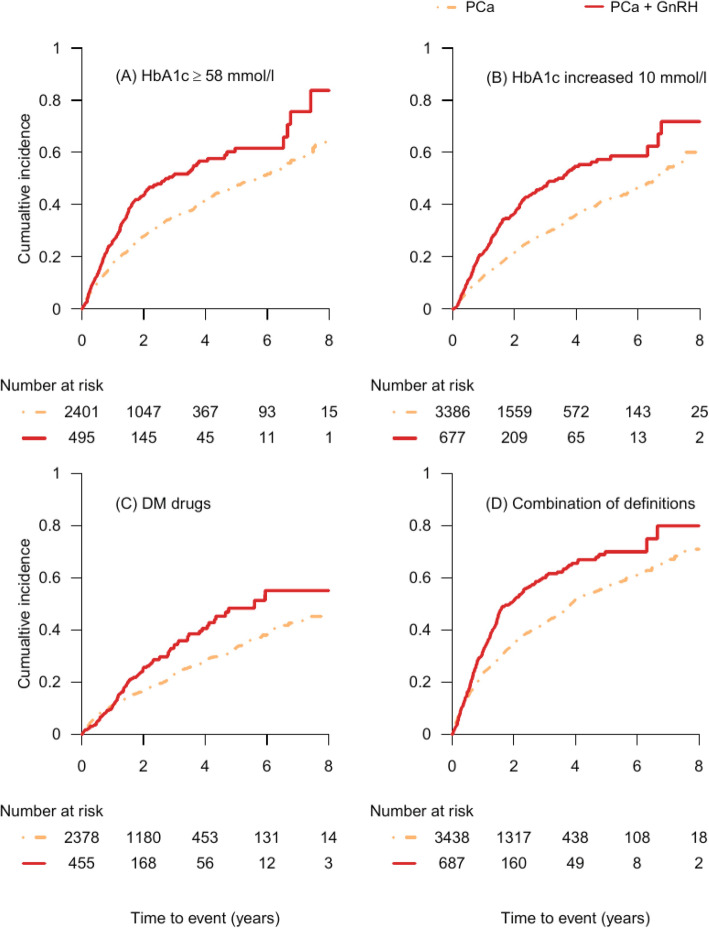
Cumulative incidence of worsening T2DM control in T2DM men by using GnRH in GnRH exposure cohort. Figure 3. Figure 3 showed Kaplan Meier Curves for cumulative incidence of worsening T2DM control in T2DM men in GnRH exposure cohort. It presented those men on GnRH had a worsening diabetes control compared with men with PCa not on GnRH over time. The changes in HbA1c measurements (Fig. 3-(a), Fig. 3-(b)) occurred earlier and more obviously than that in the addition of new antidiabetic medications (Fig. 3-(c)). Figure 3-(d) showed the cumulative incidence of combination of definitions which was combined by criteria of worsening diabetes control in Fig. 3-(a), Fig. 3-(b) and Fig. 3-(c). We observed that cumulative incidence of combination of definitions was higher in PCa men receiving GnRH compared with PCa men but not on GnRH over time. a. In Fig. 3-(a), we excluded men with a HbA1c over 58 mmol/l and men without HbA1c data at baseline. In Fig. 3-(b), men without HbA1c data at baseline were excluded. Men using insulin and men without antidiabetic drugs at the baseline were excluded in Fig. 3-(c)

Men on GnRH had a worsening diabetes control compared with men with PCa not on GnRH over time (Fig. 3). The changes in HbA1c measurements (Fig. 3-a, Fig. 3-b) occurred earlier and were more obvious than that in the addition of new antidiabetic medications (Fig. 3-c), similar to that seen in the PCa + GnRH exposure cohort.

## Discussion

In this nationwide, population-based study, use of GnRH was associated with worsening diabetes control in men with diabetes and PCa treated with GnRH.

### PCa + GnRH exposure cohort

We found that starting GnRH worsened diabetes control in T2DM men compared with men with diabetes without PCa in accordance with results of previous observational studies [3, 17]. These showed that men with PCa treated with GnRH had an increased risk of T2DM treatment escalations, compared with men with PCa not on GnRH [3] and use of GnRH increased HbA1c level and worsened diabetes control in men with PCa [17].

Several biological mechanisms have been proposed to explain the association between use of GnRH and worsening diabetes control [8]. Low levels of testosterone, induced by GnRH, are implicated in the development of insulin resistance [18, 19], which results in increased plasma glucose levels [20] and hence leads to worsening control of T2DM.

We also found that changes in HbA1c occurred prior to changes in T2DM drugs use in PCa men receiving GnRH, which is logical since antidiabetic treatment will only be changed when the deterioration of diabetes control has been verified on repeat measures.

Besides, in this cohort, we also found no statically significant association of PCa diagnosis (all risk categories) with worsening diabetes control in line with results of two previous studies [3, 21], which investigated the effect of PCa diagnosis on glycemic control and T2DM treatments. Advanced PCa, including regional metastatic and distance metastatic disease, was associated with worsening of diabetes control compared with men without PCa [3]. This finding may be explained by the use of GnRH in men with advanced PCa [4]. In addition, GnRH reduce insulin sensitivity [22], which could lead to worsening of diabetic control [23]. Therefore, their use may explain the association of worsening T2DM control with more advanced PCa.

### GnRH exposure cohort

Next, we wanted to further demonstrate that the association with worsening diabetes control was caused by the GnRH rather than the PCa diagnosis per se. In the GnRH exposure cohort, T2DM men with PCa who started GnRH after PCa diagnosis had worse diabetes control than men with PCa not on GnRH, supporting the hypothesis that it is the GnRH driving the worse control.

Notably, no association between GnRH and escalation antidiabetic drugs was found in the GnRH exposure cohort. Potentially the fact that no association was seen could represent a reluctance of clinicians to institute changes in diabetic medications in PCa men with GnRH, compared with PCa men without GnRH. Moreover, many other factors may play a role in this association in the real world, for example, the behaviours and reactions of patients and healthcare professionals, which warrants further study.

### Strengths and limitations

Our study has several strengths. First, we were able to assemble a nationwide population-based cohort of men with T2DM from the largest diabetes register in the world, with up to 10 years of follow up. To our knowledge, this is the largest population-based cohort study exploring the association of use of GnRH with diabetes control in men with T2DM. We were also able to disentangle the impact of a PCa diagnosis itself and explore the association with different risk categories of PCa. This was achieved by selecting men with T2DM with or without PCa and also with or without GnRH separately at baseline, thereby assembling two cohorts. Secondly, we were able to look at three separate markers of worsening glycaemic control due to the detailed longitudinal information within the NDR. Third, cases and relevant comparisons were matched on average time between two NDR visits. It reduced the impact of different time between visits, which is likely to be associated with quality of T2DM management. Finally, the NDR and PCBaSe 4.1 contain information on various critical confounders, including age, comorbidity, civil status, BMI, physical activity, and smoking status, which can be adjusted in the statistic models.

Limitations include that there was not sufficient power to fully explore the association of different risk categories of PCa on the worsening of T2DM control. Secondly, approximately 3–6% of men had missing data at baseline measurements. We used the last observation carried forward to impute the missing data, which may underestimate the effect of exposures. Thirdly, the data on the T2DM complications was not available for the current study. The association of GnRH and PCa diagnosis with T2DM complications warrants further study. Last, residual confounding cannot be excluded, for example, by family history of T2DM and PCa.

## Conclusions

In this large population-based cohort study, starting GnRH was associated with worsening of diabetes control in men with T2DM and PCa on GnRH compared with matched PCa-free men with T2DM, as well as compared with men with T2DM and PCa not on GnRH. Our findings highlight the need to closely monitor diabetes control in T2DM men with PCa, especially when starting on GnRH.

## Data Availability

The data that support the findings of this study are available from PCBaSe Sweden, but restrictions apply to the availability of these data, which were used under license for the current study, and so are not publicly available. Data are however available from the authors upon reasonable request and with permission of PCBaSe Sweden.

## Abbreviations

PCa: prostate cancer
GnRH: Gonadotropin Releasing Hormones agonists
T2DM: type 2 diabetes mellitus
PCBaSe: Prostate Cancer data Base Sweden
NPCR: the National Prostate Cancer Register
PDR: National Prescribed Drug Register
NDR: National Diabetes Registry
NCCN: National Comprehensive Cancer Network
HR: Hazard ratios
CI: confidence interval
CCI: Charlson Comorbidity Index
